# Patients’ demographic and socioeconomic characteristics influence the therapeutic decision-making process in psoriasis

**DOI:** 10.1371/journal.pone.0237267

**Published:** 2020-08-12

**Authors:** Emanuele Scala, Matteo Megna, Paolo Amerio, Giuseppe Argenziano, Graziella Babino, Federico Bardazzi, Luca Bianchi, Giacomo Caldarola, Anna Campanati, Serafinella Patrizia Cannavò, Andrea Chiricozzi, Andrea Conti, Giovanni Damiani, Paolo Dapavo, Clara De Simone, Maria Esposito, Gabriella Fabbrocini, Maria Concetta Fargnoli, Francesca Ferrara, Rosaria Fidanza, Giulio Gualdi, Claudio Guarneri, Katharina Hansel, Piergiorgio Malagoli, Giovanna Malara, Giuseppe Micali, Cristina Mugheddu, Maria Letizia Musumeci, Giulia Odorici, Annamaria Offidani, Leonardo Pescitelli, Francesca Prignano, Annunziata Raimondo, Simone Ribero, Franco Rongioletti, Luca Stingeni, Caterina Trifirò, Salvatore Zanframundo, Anna Balato

**Affiliations:** 1 Department of Clinical Medicine and Surgery, University of Naples Federico II, Naples, Italy; 2 Department of Medicine and Aging Science, Dermatologic Clinic, G. D'Annunzio University, Chieti-Pescara, Chieti, Italy; 3 Dermatology Unit, University of Campania “Luigi Vanvitelli”, Naples, Italy; 4 Division of Dermatology, Department of Experimental, Diagnostic and Specialty Medicine, University of Bologna, Bologna, Italy; 5 Dermatologic Unit, Department of Systems Medicine, University of Rome Tor Vergata, Rome, Italy; 6 Department of Dermatology, A. Gemelli University Hospital and Institute for Research and Cancer, IRCCS, Sacred Heart Catholic University, Rome, Italy; 7 Dermatological Clinic, Department of Clinical and Molecular Sciences, Polytechnic Marche University, Ancona, Italy; 8 Department of Clinical and Experimental Medicine, University of Messina, Messina, Italy; 9 Dermatology Unit, Department of Clinical and Experimental Medicine, University of Pisa, Pisa, Italy; 10 Department of Surgical, Medical, Dental and Morphological Sciences with Interest Transplant, Oncological and Regenerative Medicine, Dermatology Unit, University of Modena and Reggio Emilia, Modena, Italy; 11 Department of Biomedical, Surgical and Dental Sciences, University of Milan, Milan, Italy; 12 Department of Biomedical Science and Human Oncology, Second Dermatologic Clinic, University of Turin, Turin, Italy; 13 Department of Dermatology, Department of Biotechnological and Applied Clinical Sciences, University of L'Aquila, L’Aquila, Italy; 14 San Salvatore Hospital, UOSD Dermatologia, L’Aquila, Italy; 15 Department of Dermatology, ASST Spedali Civili, University of Brescia, Brescia, Italy; 16 Department of Biomedical and Dental Sciences and Morphofunctional Imaging, University of Messina, Messina, Italy; 17 Section of Dermatology, Department of Medicine, University of Perugia, Perugia, Italy; 18 Dermatology Unit, Azienda Ospedaliera San Donato Milanese, Milan, Italy; 19 Dermatology Unit, Grande Ospedale Metropolitano “Bianchi Melacrino Morelli”, Reggio Calabria, Italy; 20 Dermatology Clinic, University of Catania, Catania, Italy; 21 Dermatology Clinic, Department of Medical Sciences and Public Health, University of Cagliari, Cagliari, Italy; 22 Department of Health Sciences Section of Dermatology, University of Florence, Florence, Italy; 23 Department of Medicine, Surgery and Dentistry “Scuola Medica Salernitana”, University of Salerno, Salerno, Italy; 24 Department of Advanced Biomedical Sciences, University of Naples Federico II, Naples, Italy; Northwestern University Feinberg School of Medicine Galter Health Sciences Library, UNITED STATES

## Abstract

**Background:**

Knowledge regarding differences in care for psoriatic patients is limited. The aim of this study was to investigate factors influencing prescription of systemic treatments for patients with psoriasis with a special focus on socioeconomic factors.

**Methods and findings:**

This was a non-interventional, cross-sectional study, conducted in 18 Italian University and/or hospital centers with psoriasis-specialized units. Questionnaires evaluating demographic and socioeconomic characteristics were administered to participants. Overall, 1880 consecutive patients affected by mild-to-severe psoriasis were recruited. Univariate and multivariable logistic regression analyses of systemic therapy prescription, with a special focus on biologics, accounting for the above mentioned characteristics were performed. Our analysis showed that all analyzed patients’ characteristics were significantly associated with biological therapy compared to non-biological systemic one. Particularly, women were less likely to receive biologics than men (OR = 0.66; 95% CI, 0.57–0.77). Elderly patients (≥65 years) and subjects with a BMI ≥30 had lower odds to receive biologics respect to adults (≥35–64 years) (OR = 0.33; 95% CI, 0.25–0.40), and subjects with BMI≥25<30 (OR = 0.64; 95% CI, 0.53–0.77), respectively. Northern and Southern patients were both less likely to receive biologics than Central patients (OR = 0.75; 95% CI, 0.63–0.89, and OR = 0.56; 95% CI,0.47–0.68, respectively). Lower economic profile and never reading books were both associated with decreased odds of receiving biological therapy.

**Conclusions:**

This study shows that sex, age, comorbidities, and socioeconomic characteristics influence the prescription of systemic treatments in psoriasis, highlighting that there are still unmet needs influencing the therapeutic decision-making process that have to be addressed.

## Introduction

Psoriasis is a chronic, immune-mediated inflammatory disease affecting 2–4% of the population [1, 2]. This condition is characterized by distinct cutaneous manifestations with associated risks of systemic complications and psychological sequalae [3, 4]. Nowadays, no definitive cure exists and patients often require life-long immune-modulating therapy. Traditionally, medical treatment options have included topical agents, phototherapy and non-biological systemic therapies [4]. The development of biological agents, such as anti-tumour necrosis factor (TNF)-α, anti-interleukin (IL)-12/23, anti-IL-17 and anti-IL-23 antibodies offers a potentially safer and long-term option for patients with moderate-to-severe psoriasis [5, 6]. A substantial heterogeneity in therapeutic survival and response with biological agents has been reported [7]. However, the reasons underlying this heterogeneity remain unclear. A range of factors, such as patients’ characteristics, genetics, disease related factors, presence of comorbidities, psychological and behavioural features may all contribute to the observed response variation [8, 9].

The aim of this study was to investigate factors influencing prescription of systemic treatments for patients with psoriasis with a special focus on socioeconomic factors.

## Materials and methods

The study was approved by the Ethics Committee for Biomedical Activities ‘Carlo Romano’ of University of Naples Federico II, and conducted according to the Declaration of Helsinki principles. Protocol number is 226/13. Each participant gave written informed consent before the onset of the study. This was a non-interventional, cross-sectional, multicenter study that included adult patients with plaque psoriasis attending 18 Italian University and/or hospital centers with psoriasis-specialized units, distributed along the whole country. Italian national health system (NHS) guarantees uniform care throughout the country to warrant equal access to care for patients [10]. Medical treatment options for psoriasis like some topical agents, phototherapy, and systemic therapies including biologics are sustained by the NHS. It is to note that biological therapy can be prescribed only in public centers with psoriasis-specialized units. In Italy, there are about 100 psoriasis-specialized units, however, not all of them are active. Moreover, the number of followed patients is not equally distributed in psoriasis-specialized units across the Country. For this study, we have selected University/hospital psoriasis units (n = 18) with more than 500 patients.

The only inclusion criteria was represented by a diagnosis of mild-to-severe psoriasis performed at least in the last 6 months; age, current therapy and any comorbidities did not represent exclusion criteria. Patients were enrolled consecutively to follow-up appointments at psoriasis-specialized units, where the routine of follow-up visits is generally scheduled every 3–4 months. Study design was based on data collection obtained from patients and dermatologists. Patients were administered a questionnaire evaluating the Dermatology Life Quality Index (DLQI) and a questionnaire that explored demographic characteristics (e.g., sex, age, civil status, and residency) and socioeconomic aspects (e.g., educational level, net salary, reading books, internet use, and sport activity) (S1 File). Dermatologists were asked to fill in a medical form for each patient involved in the study exploring psoriasis-related characteristics [e.g., Psoriasis Area Surface Index (PASI), and lesion localization], pharmacological anamnesis (e.g., previous and current therapies) and other features like comorbidities, and body mass index (BMI) (S1 File). The distribution of questionnaires reflects a unique moment and no follow-up visits were scheduled for this study. As a consequence, the therapeutic decision was not affected by patients’ questionnaires. Clinical characteristics were registered at the time of the scheduled visit when the questionnaire was performed. It has to be taken into account that clinical scores did not always reflect the severity of the disease since patients could have been on treatment. A unique code was assigned to each psoriasis-specialized Unit by the coordinating center which was represented by University of Naples Federico II. The 2 patients’ questionnaires and the medical form reported the Unit code and a consecutive number referring to each patient. All data were computerized at the coordinating center. This study was conducted between September 2017 and February 2018.

### Statistical analysis

To have an adequate number of subjects, we hypothesized to obtain similar results to Naldi et al. [11] regarding educational attainment. They reported that 45% (2217 out of 4926) of the sample starting a biological treatment had an educational attainment of upper secondary school diploma (OR 1.35; 95% CI 1.12-1-62; *p*-value 0.002; with primary school as reference). We planned to include 1000 patients with plaque psoriasis to include a representative sample size (SS) in a period of 6 months. The SS was calculated according to the following formula:

$$SS=Z^{2}*(p)*\left(1-p\right)/c^{2}$$

Z value used was 1.96 considering 95% confidence level; p (percentage) value used was 0.5; c (confidence interval) value used was 0.02. We first determined the percentage of patients who currently were on topical treatment, phototherapy, systemic and biological therapy. After this, we reported the percentage of these individuals with each of the analyzed characteristics. The latter included sex, age, PASI, DLQI, psoriasis skin localization, comorbidities, BMI, previous systemic or biological therapies, civil status, educational level, net salary, region, reading books, internet use, and sport activity. We conducted univariate analysis on each of these characteristics taking into account systemic therapies as outcome variable. Categorical values were described by count and proportions. Two-sided *P*-values below 0.05 were considered significant. The multicollinearity assumption was tested using a variance inflation factor, eliminating variables with high correlation (value > 0.9), such as “newspapers reading”, “national news watching”, and “political broadcasts or debates watching”. To determine the association of each of the characteristics with the current use of systemic therapies (non-biological and biological), we constructed a multivariable logistic regression model. We adjusted and controlled for all selected variables by calculating odds ratios (ORs) and 95% confidence intervals (CIs) for each predictive variable. Multiple regression analyses were also conducted to evaluate the association of each of variables with the current class of biologics (i.e., anti-TNF-α, anti-IL-12/23, and anti-IL-17). ORs were calculated with 95% CIs. Statistical analyses were performed using SAS software v 9.3 (SAS Institute Inc., Cary, NC, USA).

## Results

### The burden of patients’ characteristics on systemic therapies

A total of 1880 psoriatic patients were recruited during the 6-month inclusion period. Out of 1880, 153 questionnaires were incomplete so our analysis was performed on 1727 subjects. Patients treated in monotherapy with topical products and phototherapy were 264 (15,2%) and 115 (6,65%), respectively. Major details are summarized in S1 Table. Overall, patients on systemic therapies were 1348 representing 70% of our sample. In view of this, we can deduce indirectly that most of enrolled subjects were affected by moderate-to-severe psoriasis. However, we cannot confirm this assumption with clinical indexes since PASI ≥10 was detected only in 448 (26%) out of 1727 patients. It has to be taken into account that all 448 patients were on treatment and in particular they were distributed as follows: 19 on topical therapies, 27 on phototherapy, 88 on non-biological systemic therapies and 314 on biologics.

Patients in monotherapy with topical treatments or phototherapy were excluded from subsequent analysis because our main purpose was to investigate factors influencing the prescription of systemic treatments (biological and non-biological ones).

Among our population, 5 subjects under the age of 18 were recruited, but none of them were on systemic therapies (S2 Table). Three hundred and sixty eight out of 1727 (368, 21.3%) patients were on treatment with non-biological systemic therapies: acitretin (n = 47), cyclosporine (n = 102), methotrexate (n = 181), and apremilast (n = 38). Nine hundred and eighty of 1727 (980, 56.7%) were on biologic treatment: anti-TNF-α [infliximab (n = 47), etanercept (n = 151), adalimumab (n = 255), certolizumab (n = 20), and golimumab (n = 12); anti-IL-12/23 [ustekinumab (n = 248)], and anti-IL-17 [secukinumab (n = 206), and ixekizumab (n = 41)].

**Fig 1:**
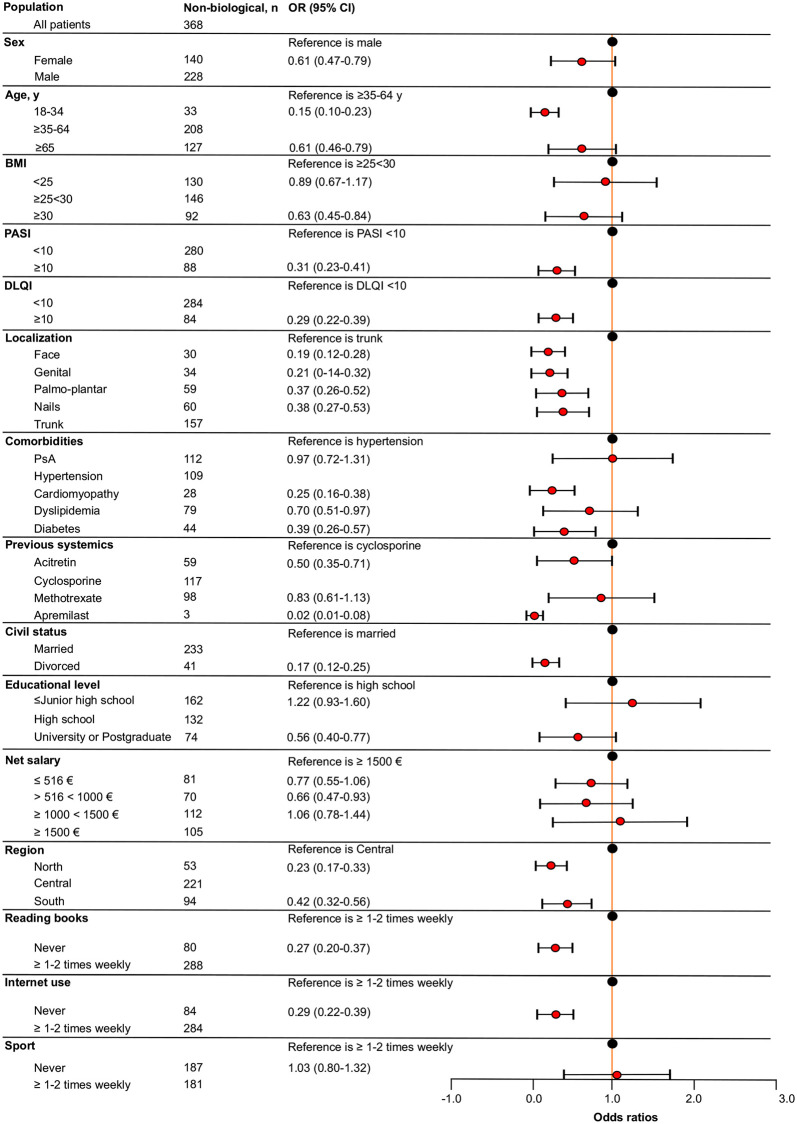
Forest plot of the fully adjusted logistic regression model to assess the association between patients’ global characteristics and non-biological therapy. Odds ratios (OR) and 95% confidence intervals (CI) are depicted. BMI, body mass index; PASI, psoriasis area severity index; DLQI, dermatologist life quality index; PsA, psoriatic arthritis.

**Fig 2:**
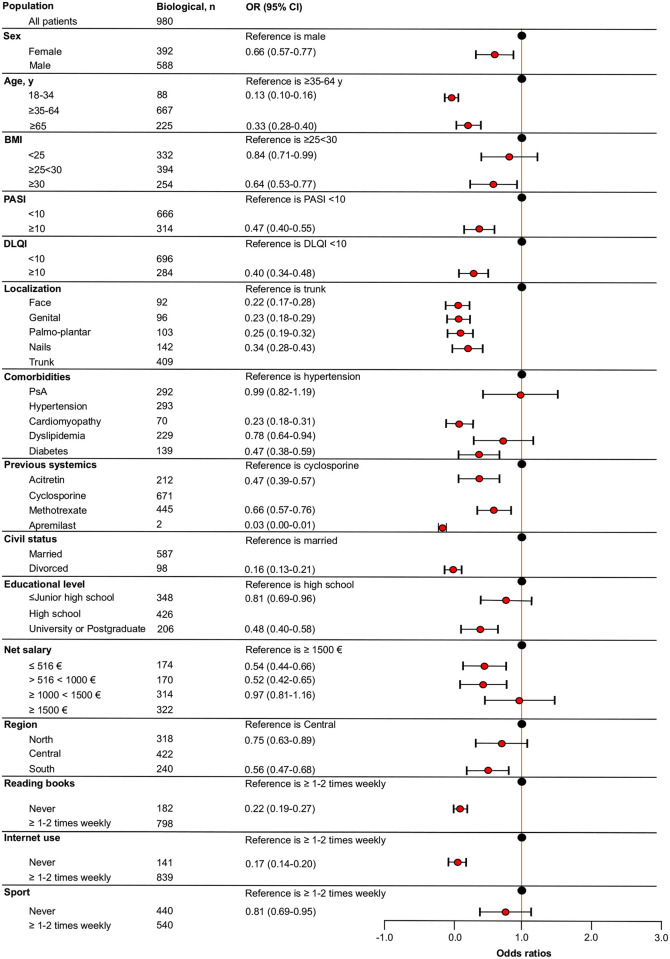
Forest plot of the fully adjusted logistic regression model to assess the association between patients’ global characteristics and biological therapy. Odds ratios (OR) and 95% confidence intervals (CI) are depicted. BMI, body mass index; PASI, psoriasis area severity index; DLQI, dermatologist life quality index; PsA, psoriatic arthritis.

Univariate analysis showed that all analyzed patients’ characteristics were significantly associated with biological therapy compared to non-biological systemic one, even though this difference was not observed for patients who had previously experienced apremilast because of limited sample size (S2 Table). As shown in Fig 1, patients with ≤ Junior high school diploma had higher odds of receiving systemic non-biological therapy compared to those with high school diploma (OR = 1.22; 95% CI, 0.93–1.60). It is to note that this was the only variable associated with this class of therapy. Considering biological therapy as outcome (Fig 2), multivariate analysis showed that females had 34% fewer odds of receiving biological therapy compared to males. Patients with an age ≥18< 34 or ≥ 65 years had lower odds for prescription of biologics compared to patients with an age ≥35<64 years. Patients with a BMI <25 and ≥30 were less associated with biological treatment respect to patients with a BMI ≥25<30. Patients with PASI and DLQI ≥10 had lower odds of receiving biologics respect to those with PASI and a DLQI <10. Subjects with a psoriasis localization at face, genital, palmo-plantar, or nails were less associated with biological therapy compared to those with a psoriasis localization at trunk. Regards comorbidities, patients with cardiomyopathy, dyslipidema and diabetes had lower odds for prescription of biologics compared to patients with hypertension. No substantial difference was encountered between patients suffering from PsA and hypertension. Among previous systemic non-biological therapies, patients who experienced acitretin, methotrexate or apremilast had lower odds of prescription for biologics compared to patients who were previously treated with cyclosporine. Concerning civil status, divorced patients were less associated with biological treatment compared to married patients.

Regards socioeconomic status, patients with lower educational attainment (≤ Junior high school) or higher educational level (University or Postgraduate) had lower odds of receiving biologics compared to patients with a high school diploma. Patients perceiving a net salary of ≤516 € or >516<1000 € per month were less associated with biologics compared to those perceiving ≥ 1500 € per month. Additionally, patients with a net salary ≥1000 <1500€ had only 3% fewer odds of receiving biological treatment respect to patients with a net salary ≥ 1500 €.

Concerning biological therapy distribution across the country, patients living in Northern and Southern Italy had 25% and 44% fewer odds, respectively, of receiving biologics respect to those living in Central Italy. Lastly, we encountered that patients who never read books, never use internet or never practice sporting activity were less associated with the prescription of biological therapy. In particular, patients who never read books had 88% fewer odds of receiving biologics compared to patients who read books (≥1 or 2 times weekly), whereas patients who never use internet had 83% fewer odds of prescription of biological treatment compared to patients who use internet (≥1 or 2 times weekly). Patients who never practice sporting activity had 19% fewer odds of receiving biologics compared to those performing sporting activity (≥1 or 2 times weekly). ORs and CIs calculated are detailed in Fig 2.

### The burden of patients’ socioeconomic characteristics on the choice of different biologics

To evaluate the association of socioeconomic variables with the current class of biologics (e.g., anti-TNF-α, anti-IL-12/23, and anti-IL-17), a multiple regression analysis was performed. Overall, 485/980 (48.9%) patients were on anti-TNF-α agents, 248/980 (25.3%) on anti-IL-12/23, and 247/980 (28.2%) on anti-IL-17 ones.

**Fig 3:**
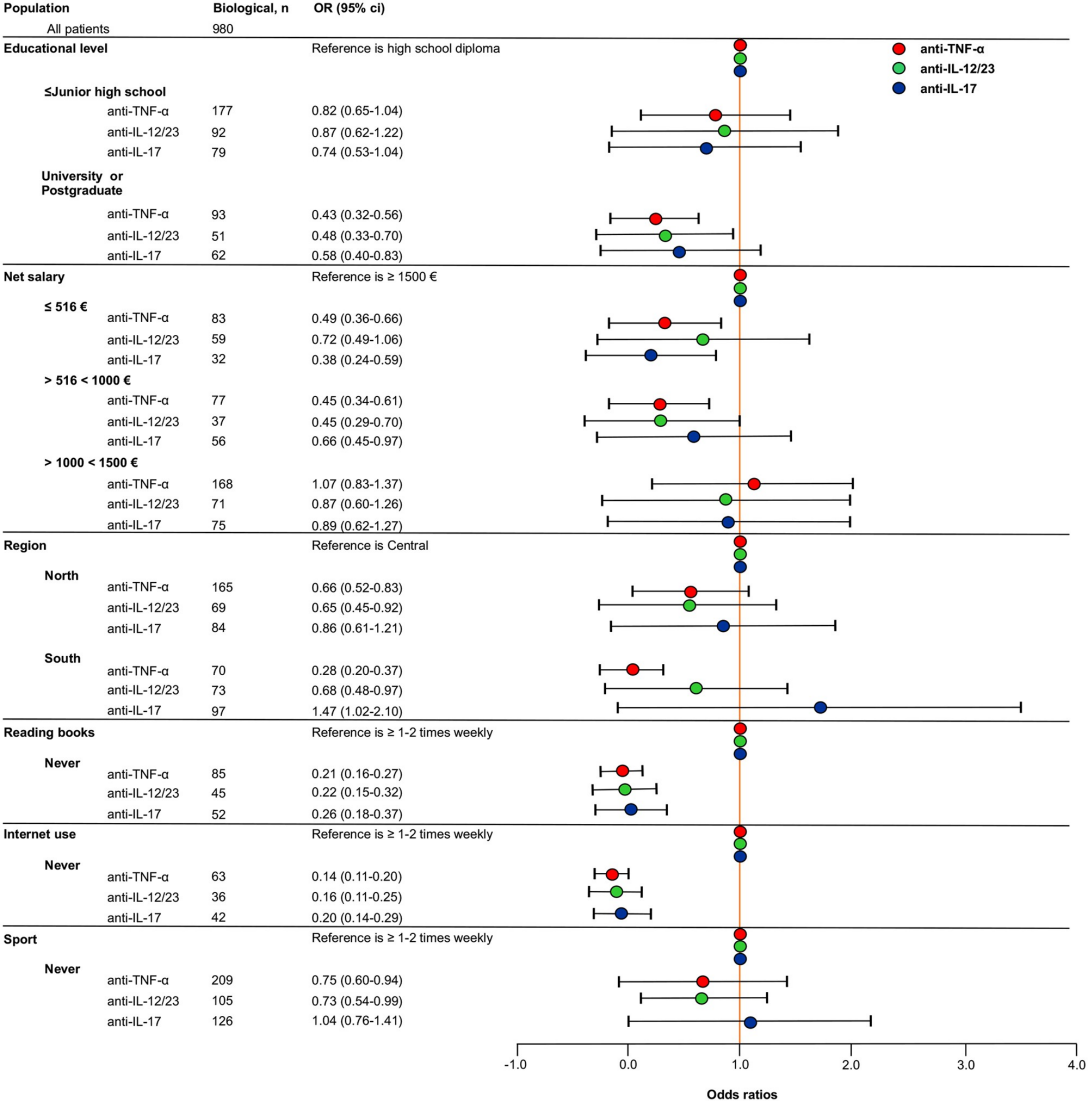
Forest plot of the fully adjusted logistic regression model to assess the association between patients’ socioeconomic characteristics and different biologics. Odds ratios (OR) and 95% confidence intervals (CI) are depicted. IL, interleukin; TNF, tumor necrosis factor.

As reported in Fig 3, patients with an educational level ≤ Junior high school had 18%, 13% and 26% fewer odds of receiving anti-TNF-α, anti-IL-12/23, and anti-IL-17 therapy respectively compared to patients with a high school diploma.

Patients with higher educational attainment (University or postgraduate) had 57%, 52% and 42% fewer odds of receiving anti-TNF-α, anti-IL-12/23, and anti-IL-17 therapy respectively compared to patients with a high school diploma.

Patients perceiving a net salary ≤ 516 € had 51%, 28% and 62% fewer odds of receiving anti-TNF-α, anti-IL-12/23, and anti-IL-17 therapy respectively compared to patients with a net salary ≥ 1500 €.

Patients perceiving a net salary >516 <1000 € had 55%, 55% and 44% fewer odds of receiving anti-TNF-α, anti-IL-12/23, and anti-IL-17 therapy respectively compared to patients with a net salary ≥ 1500 €.

Patients perceiving a net salary ≥1000 <1500 € had 13% and 11% fewer odds of receiving anti-IL-12/23, and anti-IL-17 therapy respectively compared to patients with a net salary ≥ 1500 €. No substantial difference was encountered for the prescription of anti-TNF-α therapy between patients perceiving ≥1000 <1500 € and ≥ 1500 €.

Concerning the distribution of biological therapies across the country, patients living in Northern Italy had 44%, 45% and 14% fewer odds of receiving anti-TNF-α, anti-IL-12/23 and anti-IL-17 therapy respectively compared to patients living in Central Italy.

Patients living in Southern Italy had 72% and 32% fewer odds of receiving anti-TNF-α and anti-IL-12/23 respectively compared to patients living in Central Italy. Conversely, they were more associated with the use of anti-IL-17 therapy respect to patients treated in Central Italy.

Among patients’ cultural profile, those who never read books had 79%, 78% and 74% fewer odds of receiving anti-TNF-α, anti-IL-12/23 and anti-IL-17 therapy respectively compared to patients who read books ≥ 1–2 times weekly.

Patients who never use internet had 86%, 84% and 80% fewer odds of receiving anti-TNF-α, anti-IL-12/23 and anti-IL-17 therapy respectively compared to patients who use internet ≥ 1–2 times weekly.

Regards sporting activity, patients who never perform sport had 25% and 27% fewer odds of receiving anti-TNF-α and anti-IL-12/23, respectively compared to patients who practice sporting activity ≥ 1–2 times weekly. No substantial difference was encountered for the prescription of anti-IL-17 therapy between patients who never perform sporting activity compared to those who practice sporting activity ≥ 1–2 times weekly.

## Discussion

Finding the most effective treatment for patients with severe psoriasis can be challenging and often entails multiple treatment attempts resulting in prolonged suffering and unnecessary medication costs. In this study, we documented the association of global patients’ characteristics with the current systemic treatments in psoriasis in order to identify factors that influence therapeutic decision. In 2009, Naldi et al. [11]. reported difference in access to biological treatments for psoriasis in Italy. In their analysis, sex did not constitute a variable associated with biological drug prescription. Here, we found that women were less likely to receive biological medications than men, as previously shown in a Swedish study [12]. It has been hypothesized that women are more likely to believe that they can influence their disease themselves and how manage it [13]. In addition, biological therapies might be considered more dangerous for young women of childbearing age, although teratogenic effects are not associated with biologics [14, 15].

With respect to patients’ age, both younger (≥18<34 years) and elderly (≥65 years) were less associated with biological therapy. It has been reported that younger psoriatic patients live more negatively psoriasis skin impairments, they are less compliant to drug treatments, and more concerned about the possible negative effects on fertility [16–19]. In particular, Umar *et al*. [19] have reported that younger patients and women were more concerned with biological treatment than older patients and men. Regarding aged population, our evidence is in line with Naldi *et al*. [11]. This could be explained by the fact that limited data are available in the literature regarding the safety of biologics in elderly psoriatic patients [20–23]. Moreover, aged patients are largely underrepresented in the clinical trials [20]. However, the recent Italian guidelines on psoriatic systemic treatments recommends that all therapy, including biologics, can be safely used in elderly psoriatic patients [24].

Normal weight patients (BMI <25) and obese ones (BMI ≥30) were less associated with biological therapy. However, patients with a BMI <25 had only 16% fewer odds of receiving biologics compared to those with a BMI ≥25<30, showing a slight difference in biologic prescription between these bodyweight categories. Conversely, obese patients had 36% of fewer odds of receiving biological treatments compared to patients with a BMI ≥25<30. In accordance with Naldi [11], a higher body mass index (BMI>30) was less associated with biological therapy. A higher BMI does not represent a negative variable on the patient’s short-term ustekinumab response according to Xie and colleagues [8], whereas according to Papp *et al*. obesity affects therapeutic response to ustekinumab [25]. Negative effects of increased BMI on clinical response were also reported for adalimumab [26]. Regarding anti-IL-17 therapy the effectiveness of treatment was similar across bodyweight categories either for ixekizumab or secukinumab, even if a lower trend of PASI 90 and PASI 100 responses was detected in obese patients [27–29]. It is not surprising that we found PASI and DLQI scores <10 mostly associated with biological drugs since all patients were on treatment. Psoriasis localizations diverse from trunk were all less associated with biologics. Face, genital, palms and nails have been labeled as ‘difficult locations’ of psoriasis. They are usually too sensitive to be treated with potent topical products for long periods, frequently necessitating systemic drugs, possibly are more resistant to biological therapies respect to other body sites and tend to show recurrent disease relapse [30]. Despite the literature is enriching of trials and real-life studies specifically investigating on biological antipsoriatic drugs’ effectiveness in difficult-to-treat areas [30, 31], our data suggest that there is still resistance on the part of dermatologists to treat psoriasis in certain body regions with biologics.

In presence of comorbidities, biological therapy was less associated with patients affected by cardiomyopathy, dyslipidemia, and diabetes respect to patients with hypertension, showing that these variables still influence therapeutic decision making. In 2003, Chung *et al*. [32] showed that anti-TNF-α therapy was associated with an increased incidence of death in patients with moderate-to-severe heart failure (New York Heart Association [NYHA] Functional Class III/IV). Nowadays, evidence from the literature suggests that anti-TNF-α therapy might be helpful to prevent cardiovascular risk in psoriatic patients [6]. Regarding the newer interleukins blockers such as anti-IL-12/23 and anti-IL-17, early data have suggested no increased cardiovascular risk [6]. However, because of the follow-up short duration, many of these results should be interpreted with caution.

Transitioning from conventional systemic therapies to biological medications is not infrequent for various reasons [33]. Practical guidance on treatment optimization and transitioning for moderate-to-severe plaque psoriasis recommends that cyclosporine should be used for short periods due to its toxicity [33]. Indeed, we found that cyclosporine experienced-patients were mostly associated with current biological therapy, highlighting that these patients mostly undergo to transitioning conventional systemic therapy to biological one. Regards small-molecule use, it is to note that only multi-failure and/or contraindicated patients to biologics can be addressed to apremilast in Italy. Thus, apremilast experienced-patients generally do not transit to biological therapy.

Concerning socioeconomic aspects, Naldi *et al*. [11] documented that marital status did not constitute a predictive variable associated with biological drug prescriptions, with no significant difference between married and divorced people. Therefore, it was unexpected that divorced patients resulted to be less associated with biologics access in our study, probably due to a small sample size of these patients.

In line with Naldi *et al*. [11], our study showed that patients with a lower level of education were less associated with biologics. Indeed, they resulted to be more associated with systemic non biological treatments. Moreover, we found that patients with lower educational level (≤ Junior high school) were mostly associated with anti-IL-12/23 therapy, whereas patients with a higher attainment than high school diploma (University and/or Postgraduate) with anti-IL-17 one. It is very hard to try to elucidate these points. A hypothesis might be represented by the fact that better negotiation skills as well as increased empathy with dermatologists can explain why patients with higher educational level are associated with newer biological therapies.

People with a lower income level had lower odds of receiving biologics than those with a net salary ≥ 1500 €. Likewise, Naldi *et al*. [11] documented a higher number of prescriptions for biologics in higher socioeconomics sectors of the Italian population. Additionally, we found that increasing the income there was no substantial difference in the prescription of the 3 analyzed classes of biologics. Differences were also observed regarding the geographical area. Central region was mostly associated with access to biologics for psoriasis, probably due to the higher number of subjects involved in this group. It is to highlight that Northern and Southern regions were mostly associated with IL-17 blockers. Regional differences in the prescription of biologics for psoriasis have been also reported in a Swedish study by Calara *et al*. [34]. In particular, the authors reported that there were significant and persistent regional differences in biologics prescription for psoriasis, also after adjusting for patients' characteristics and standard measures of disease severity. Although the Swedish healthcare system has several measures against inequitable treatment access, the same authors conclude that treatment options for psoriasis depend on where care is received.

Patients who are not used to read books or surf in internet were less associated with biological therapies and a similar distribution among the different biologics was observed. We wondered if these variables might influence the therapeutic decision-making process in psoriasis. We hypothesize that informed patients might express their opinion as regards treatments, preferring the newer biological therapies. Indeed, Umar *et al*. reported that a closer match between physicians’ recommendations and patients’ preferences is associated with greater treatment adherence and satisfaction by patients who influence, in part, the therapeutic decision-making process. In particular, patients express their preference on treatment duration, treatment frequency, treatment location, probability of side effects, and reversibility of side effects [35].

Regarding sporting activity, the latter did not constitute a variable tightly associated with biological drug prescription, since patients who never practice sporting activity had only 19% (OR = 0.81) fewer odds of receiving biologics compared to those performing sporting activity (≥1 or 2 times weekly). Although it has been shown that regular physical activity has a beneficial effect on the natural course of the disease [36] and may lower the risk of psoriasis onset [37], the dermatologists appear to be not influenced by this variable during therapeutic decision.

## Limitations

The findings of this study have to be seen in light of some limitations. All patients were ongoing therapy, and surveys before starting treatments would have been preferable as screening for therapeutic choice. It should be noted that the decision to start biological treatment is made by the dermatologist in dialogue with the patient, and this process was not registered. We can therefore not exclude a potential bias deriving from this undocumented dialogue. Another limitation derived from the fact that the survey was performed in a unique moment and only in follow-up patients is the unavailability of reliable disease severity. Clinical indexes did not reflect the severity of psoriasis for ongoing therapy. Our interest was to uncover if demographic and socioeconomic characteristics of psoriatic patients treated in different regions of our Country might influence the prescription of systemic therapies. Notable regional differences were found, but the different extent of geographical regions, the non uniform distribution of psoriasis-specialized units across Northern, Central, and Southern Italy and differences in regional self-government represent another study limitation.

## Conclusions

This study shows that sex, age, comorbidities, and socioeconomic characteristics influence the prescription of systemic treatments in psoriasis, highlighting that there are still unmet needs influencing the therapeutic decision-making process that have to be addressed. Further studies, especially with a case-control design, are required to deeper investigate this topic.

## Supporting information

S1 Table Patients’ characteristics on non-systemic therapies. (DOCX)

S2 Table Patients’ characteristics on systemic therapies. Univariate analysis.
(DOCX)

S1 File Questionnaire. Evaluation of demographic and socioeconomic characteristics of psoriatic patients (English version).
(PDF)

S2 File Questionnaire. Evaluation of demographic and socioeconomic characteristics of psoriatic patients (Italian version).
(PDF)
