# Supplementary material for: Patients’ demographic and socioeconomic characteristics influence the therapeutic decision-making process in psoriasis
Source: PLoS One. 2020 Aug 12;15(8):e0237267. doi: 10.1371/journal.pone.0237267 (PMC7423114; doi:10.1371/journal.pone.0237267)
Supplement: S1 Table — (DOCX) [file pone.0237267.s001.docx]

| **Population** | **All patients, n** | **Topical therapy, n (%)** | **Phototherapy, n (%)** |
| --- | --- | --- | --- |
| Patients | 1727 | 264 | 115 |
| **Sex** |  |  |  |
| Female | 681 | 98 (15) | 51 (7) |
| Male | 1046 | 166 (16) | 64 (6) |
| **Age, y** |  |  |  |
| < 18 | 5 | 3 (60) | 2 (40) |
| 18-34 | 190 | 43 (23) | 26 (14) |
| 35-64 | 1087 | 146 (14) | 66 (6) |
| ≥ 65 | 445 | 72 (16) | 21 (5) |
| **BMI** |  |  |  |
| <25 | 652 | 129 (20) | 61 (9) |
| ≥25 <30 | 667 | 88 (13) | 39 (6) |
| ≥30 | 408 | 47 (12) | 15 (4) |
| **PASI** |  |  |  |
| <10 | 1279 | 245 (19) | 88 (7) |
| ≥10 | 448 | 19 (4) | 27 (6) |
| **DLQI** |  |  |  |
| <10 | 1295 | 235 (18) | 80 (6) |
| ≥10 | 432 | 29 (7) | 35 (8) |
| **Localization** |  |  |  |
| Face | 163 | 23 (14) | 18 (11) |
| Genital | 174 | 30 (17) | 14 (8) |
| Palmo-plantar | 216 | 31 (15) | 18 (8) |
| Nails | 253 | 32 (13) | 19 (8) |
| Trunk | 713 | 87 (12) | 60 (8) |
| **Comorbidities** |  |  |  |
| PsA | 411 | 6 (1) | 1 (0.2) |
| Hypertension | 481 | 60 (12) | 19 (4) |
| Cardiomyopathy | 128 | 22 (17) | 8 (6) |
| Dyslipidemia | 350 | 30 (9) | 12 (3) |
| Diabetes | 213 | 23 (11) | 7 (3) |
| **Previous systemic therapies** |  |  |  |
| Acitretin | 280 | 7 (3) | 2 (0.7) |
| Cyclosporine | 838 | 33 (4) | 17 (2) |
| Methotrexate | 549 | 5 (1) | 1 (0.1) |
| Apremilast | 5 | 0 (0) | 0 (0) |
| **Civil status** |  |  |  |
| Married | 1042 | 163 (16) | 59 (6) |
| Divorced | 166 | 17 (10) | 10 (6) |
| **Educational level** |  |  |  |
| ≤ Junior high school | 626 | 85 (14) | 31 (5) |
| High school | 726 | 125 (17) | 43 (6) |
| University or Postgraduate | 375 | 54 (14) | 41 (11) |
| **Net salary** |  |  |  |
| ≤ 516 € | 307 | 17 (6) | 35 (11) |
| > 516 < 1000 € | 291 | 40 (14) | 11 (4) |
| ≥ 1000 < 1500 € | 533 | 76 (14) | 31 (6) |
| ≥ 1500 € | 596 | 131 (22) | 38 (6) |
| **Region** |  |  |  |
| North | 378 | 2 (0.5) | 5 (1) |
| Central | 925 | 227 (25) | 55 (6) |
| South | 424 | 35 (8) | 55 (13) |
| **Reading books** |  |  |  |
| Never | 341 | 53 (16) | 26 (8) |
| ≥ 1-2 times weekly | 1386 | 211 (15) | 89 (6) |
| **Internet use** |  |  |  |
| Never | 285 | 48 (17) | 12 (4) |
| ≥ 1-2 times weekly | 1442 | 216 (15) | 103 (7) |
| **Sport** |  |  |  |
| Never | 772 | 101 (13) | 44 (6) |
| ≥ 1-2 times weekly | 955 | 163 (17) | 71 (7) |

**Table S1.** Patients’ characteristics on non-systemic therapies.
