# Supplementary material for: Patients’ demographic and socioeconomic characteristics influence the therapeutic decision-making process in psoriasis
Source: PLoS One. 2020 Aug 12;15(8):e0237267. doi: 10.1371/journal.pone.0237267 (PMC7423114; doi:10.1371/journal.pone.0237267)
Supplement: S2 Table — Univariate analysis. (DOCX) [file pone.0237267.s002.docx]

| **Table S2. Patients’ characteristics on systemic therapies.** Univariate analysis. | | | | |
| --- | --- | --- | --- | --- |
|  | | | | |
| **Population** | **All patients, n** | **Non-biological Systemic therapy, n (%)** | **Biological therapy, n (%)** | ***p*-value** |
|  | 1727 | 368 | 980 |  |
| **Sex** |  |  |  |  |
| Female | 681 | 140 (20) | 392 (58) | < 0.001 |
| Male | 1046 | 228 (22) | 588 (56) | < 0.001 |
| **Age, y** |  |  |  |  |
| < 18 | 5 | 0 (0) | 0 (0) | NA |
| ≥18≤34 | 190 | 33 (17) | 88 (46) | < 0.001 |
| ≥35≤64 | 1087 | 208 (19) | 667 (61) | < 0.001 |
| ≥ 65 | 445 | 127 (28) | 225 (51) | < 0.001 |
| **BMI** |  |  |  |  |
| <25 | 652 | 130 (20) | 332 (51) | < 0.001 |
| ≥25 <30 | 667 | 146 (22) | 394 (59) | < 0.001 |
| ≥30 | 408 | 92 (22) | 254 (62) | < 0.001 |
| **PASI** |  |  |  |  |
| <10 | 1279 | 280 (22) | 666 (52) | < 0.001 |
| ≥10 | 448 | 88 (20) | 314 (70) | < 0.001 |
| **DLQI** |  |  |  |  |
| <10 | 1295 | 284 (22) | 696 (54) | < 0.001 |
| ≥10 | 432 | 84 (19) | 284 (65) | < 0.001 |
| **Localization** |  |  |  |  |
| Face | 163 | 30 (18) | 92 (56) | < 0.001 |
| Genital | 174 | 34 (19) | 96 (55) | < 0.001 |
| Palmo-plantar | 216 | 59 (27) | 103 (48) | < 0.001 |
| Nails | 253 | 60 (24) | 142 (56) | < 0.001 |
| Trunk | 713 | 157 (22) | 409 (57) | < 0.001 |
| **Comorbidities** |  |  |  |  |
| PsA | 411 | 112 (27) | 292 (71) | < 0.001 |
| Hypertension | 481 | 109 (23) | 293 (61) | < 0.001 |
| Cardiomyopathy | 128 | 28 (22) | 70 (55) | < 0.001 |
| Dyslipidemia | 350 | 79 (23) | 229 (65) | < 0.001 |
| Diabetes | 213 | 44 (21) | 139 (65) | < 0.001 |
| **Previous non-biological systemic therapies** |  |  |  |  |
| Acitretin | 280 | 59 (21) | 212 (76) | < 0.001 |
| Cyclosporine | 838 | 117 (14) | 671 (80) | < 0.001 |
| Methotrexate | 549 | 98 (18) | 445 (81) | < 0.001 |
| Apremilast | 5 | 3 (60) | 2 (40) | NS |
| **Civil status** |  |  |  |  |
| Married | 1042 | 233 (22) | 587 (56) | < 0.001 |
| Divorced | 166 | 41 (25) | 98 (59) | < 0.001 |
| **Educational level** |  |  |  |  |
| ≤ Junior high school | 626 | 162 (26) | 348 (56) | < 0.001 |
| High school | 726 | 132 (18) | 426 (59) | < 0.001 |
| University or Postgraduate | 375 | 74 (20) | 206 (55) | < 0.001 |
| **Net salary** |  |  |  |  |
| ≤ 516 € | 307 | 81 (26) | 174 (57) | < 0.001 |
| > 516 < 1000 € | 291 | 70 (24) | 170 (58) | < 0.001 |
| ≥ 1000 < 1500 € | 533 | 112 (21) | 314 (59) | < 0.001 |
| ≥ 1500 € | 596 | 105 (18) | 322 (54) | < 0.001 |
| **Region** |  |  |  |  |
| North | 378 | 53 (14) | 318 (84) | < 0.001 |
| Central | 925 | 221 (24) | 422 (46) | < 0.001 |
| South | 424 | 94 (22) | 240 (57) | < 0.001 |
| **Reading books** |  |  |  |  |
| Never | 341 | 80 (23) | 182 (53) | < 0.001 |
| ≥ 1-2 times weekly | 1386 | 288 (21) | 798 (58) | < 0.001 |
| **Internet use** |  |  |  |  |
| Never | 285 | 84 (29) | 141 (49) | < 0.001 |
| ≥ 1-2 times weekly | 1442 | 284 (20) | 839 (58) | < 0.001 |
| **Sport** |  |  |  |  |
| Never | 772 | 187 (24) | 440 (57) | < 0.001 |
| ≥ 1-2 times weekly | 955 | 181 (19) | 540 (56) | < 0.001 |
| The percentages are calculated as a function of the number of patients in the group described in row. BMI, body mass index; PASI, psoriasis area severity index; DLQI, dermatologist life quality index; PsA, psoriatic arthritis; NA, not applicable; NS, not significant. | | | | |
