# Supplementary material for: Patients’ demographic and socioeconomic characteristics influence the therapeutic decision-making process in psoriasis
Source: PLoS One. 2020 Aug 12;15(8):e0237267. doi: 10.1371/journal.pone.0237267 (PMC7423114; doi:10.1371/journal.pone.0237267)
Supplement: S1 File — Evaluation of demographic and socioeconomic characteristics of psoriatic patients (English version). (PDF) [file pone.0237267.s003.pdf]

Unit code ☐☐

Patient number ☐☐☐

<u>MEDICAL FORM</u> *(filled in by the dermatologist)*

Last name (only the first three letters) ☐☐☐

Name (only the first three letters) ☐☐☐

Date of birth: ____/____/_______ **Place of birth**:____________________ F □ M □

Height (cm): ☐☐☐

Weight (kg): ☐☐☐

BMI: ☐☐☐

PASI:___________ **BSA**(%):_________ **DLQI**:_________

Psoriasis skin lesion localization:

- □ Scalp
- □ Face
- □ Trunk
- □ upper limbs
- □ lower limbs
- □ genital region
- □ skin folds
- □ palmo-plantar region
- □ nails

Psoriasis clinical form:

- □ Plaque
- □ Guttate
- □ Pustular
- □ Erythrodermic

Psoriasis year of onset: ☐☐☐☐

Psoriatic arthritis: ◻ no ◻ yes

Year of psoriatic arthritis onset: | | | | |

Comorbidities:

- ◻ Diabetes
- ◻ Dyslipidemia
- ◻ Hypertension
- ◻ Ischemic Cardiomyopathy
- ◻ Other (specify) …………………………………………………………………………

Previous psoriasis therapies: ◻ no ◻ yes (if yes, please tick which of the following ones)

- ◻ Topical agents
- ◻ Phototherapy
- ◻ Systemic agents: ◻ acitretin
  ◻ ciclosporin
  ◻ methotrexate
  ◻ apremilast
- ◻ Biologics: ◻ efalizumab
  ◻ infliximab
  ◻ adalimumab
  ◻ etanercept
  ◻ golimumab
  ◻ certolizumab pegol
  ◻ ustekinumab
  ◻ secukinumab
  ◻ ixekizumab

Current therapy: ◻ no ◻ yes (if yes, please tick which of the following ones)

- ◻ Topical agents
- ◻ Phototherapy
- ◻ Systemic agents: ◻ acitretin
  ◻ ciclosporin
  ◻ methotrexate
  ◻ apremilast
- ◻ Biologics: ◻ infliximab
  ◻ adalimumab
  ◻ etanercept
  ◻ golimumab
  ◻ certolizumab pegol
  ◻ ustekinumab
  ◻ secukinumab
  ◻ ixekizumab

Unit code | | |

Patient number | | | |

PATIENT FORM *(filled in by the patient)*

Last name (only the first three letters) **Name** (only the first three letters)

Civil status:

- ☐ unmarried
- ☐ married
- ☐ cohabitant
- ☐ divorced / separated
- ☐ widowed

Education qualification

- ☐ none
- ☐ primary school diploma
- ☐ junior high school diploma
- ☐ high school diploma
- ☐ University degree
- ☐ post graduation

Which is your current professional status?

- ☐ Office worker
- ☐ Laborer
- ☐ Student
- ☐ Housekeeper
- ☐ Unemployed
- ☐ Retired
- ☐ Family business owner or helper
- ☐ Freelancer
- ☐ Individual entrepreneur
- ☐ Looking for the first job
- ☐ Manager
- ☐ Managerial employee
- ☐ Partner of companies
- ☐ Occasional worker
- ☐ Self-employed worker (artist, artisan, retailer)
- ☐ Unregistered worker

Approximately, which of the following classes does your monthly income belong to?

- □ none
- □ less than € 516
- □ from € 516 to € 1000
- □ from € 1001 to € 1500
- □ more than € 1500

How often do you practice the following activities ?

Newspapers reading:	□ never	□ rarely	□sometimes	□ often
National news watching:	□ never	□ rarely	□sometimes	□ often
Political broadcasts or debates watching:	□ never	□ rarely	□sometimes	□ often

How often do you practice the following activities weekly?

Going to the cinema	□ never	□ once or twice	□ more than twice
Practicing some sports (indoor or outdoor)	□ never	□ once or twice	□ more than twice
Volunteering	□ never	□ once or twice	□ more than twice
Reading (Books)	□ never	□ once or twice	□ more than twice
Watching TV	□ never	□ once or twice	□ more than twice
Surfing internet	□ never	□ once or twice	□ more than twice
Leisure manual activities	□ never	□ once or twice	□ more than twice
(playing musical instruments, painting)			
