# Supplementary material for: Patients’ demographic and socioeconomic characteristics influence the therapeutic decision-making process in psoriasis
Source: PLoS One. 2020 Aug 12;15(8):e0237267. doi: 10.1371/journal.pone.0237267 (PMC7423114; doi:10.1371/journal.pone.0237267)
Supplement: S2 File — Evaluation of demographic and socioeconomic characteristics of psoriatic patients (Italian version). (PDF) [file pone.0237267.s004.pdf]

Codice unità ☐☐

Numero paziente ☐☐☐

SCHEDA MEDICA *(compilata da parte del dermatologo)*

Cognome (solo le prime tre lettere) ☐☐☐

Nome (solo le prime tre lettere) ☐☐☐

Data di nascita: ____/____/_______ **Luogo di nascita**:___________________ F □ M □

Altezza (cm): ☐☐☐

Peso (kg): ☐☐☐

BMI: ☐☐☐

PASI:__________ **BSA**(%):_________ **DLQI**:_________

Localizzazione della psoriasi:

- □ Scalpo
- □ Faccia
- □ Tronco
- □ Arti superiori
- □ Arti inferiori
- □ Regione genitale
- □ Pieghe delle pelle
- □ Regione palmo-plantare
- □ Unghie

Forme cliniche di psoriasi:

- □ Placche
- □ Guttata
- □ Pustolosa
- □ Eritrodermica

Anno di insorgenza della psoriasi: ☐☐☐☐

Artrite psoriasica: □ no □ si

Anno di insorgenza dell'artrite psoriasica: | | | | |

Comorbidità:

- □ Diabete
- □ Dislipidemia
- □ Ipertensione
- □ Cardiopatia ischemica
- □ Altre (specificare) ………………………………………………………………………………

Precedenti terapie per la psoriasi: □ no □ si (se si, barrare quale tra le seguenti)

- □ Agenti topici
- □ Fototerapia
- □ Terapia sistemica: □ acitretina
  □ ciclosporina
  □ metotressato
  □ apremilast
- □ Biologici: □ efalizumab
  □ infliximab
  □ adalimumab
  □ etanercept
  □ golimumab
  □ certolizumab pegol
  □ ustekinumab
  □ secukinumab
  □ ixekizumab

Terapia attuale: □ no □ si (se si, barrare quale tra le seguenti)

- □ Agenti topici
- □ Fototerapia
- □ Terapia sistemica: □ acitretina
  □ ciclosporina
  □ metotressato
  □ apremilast
- □ Biologici: □ infliximab
  □ adalimumab
  □ etanercept
  □ golimumab
  □ certolizumab pegol
  □ ustekinumab
  □ secukinumab
  □ ixekizumab

Codice Unità | | |

Numero paziente | | | |

SCHEDA PAZIENTE *(compilata da parte del paziente)*

Cognome (solo le prime tre lettere) **Nome** (solo le prime tre lettere)

Stato civile:

- ◻ non sposato
- ◻ sposato
- ◻ convivente
- ◻ divorziato / separato
- ◻ vedovo

Titolo di studio

- ◻ nessuno
- ◻ Licenza elementare
- ◻ Licenza media
- ◻ Diploma di scuola superiore
- ◻ Laurea
- ◻ Post laurea

Qual è il tuo attuale stato professionale?

- ◻ Impiegato
- ◻ Lavoratore
- ◻ Studente
- ◻ Casalinga
- ◻ Disoccupato
- ◻ Pensionato
- ◻ Proprietario o aiutante dell'azienda familiare
- ◻ Libero professionista
- ◻ Imprenditore individuale
- ◻ Alla ricerca del primo lavoro
- ◻ Dirigente
- ◻ Impiegato direttivo
- ◻ Partner di aziende
- ◻ Lavoratore occasionale
- ◻ Lavoratore autonomo (artista, artigiano, venditore)
- ◻ Lavoratore non registrato

Approssimativamente, a quale delle seguenti classi appartiene il tuo reddito mensile?

- □ nessuno
- □ meno di € 516
- □ da € 516 a € 1000
- □ da € 1001 a € 1500
- □ più di € 1500

Con quale frequenza pratichi le seguenti attività?

Lettura di quotidiani:	□ mai	□ raramente	□ a volte	□ spesso
Notiziari nazionali (TV):	□ mai	□ raramente	□ a volte	□ spesso
Trasmissioni politiche o dibattici (TV):	□ mai	□ raramente	□ a volte	□ spesso

Con quale frequenza pratichi settimanalmente le seguenti attività?

Andare al cinema	□ mai	□ uno o due volte	□ più di due volte
Praticare quale sport (interno o all'esterno)	□ mai	□ uno o due volte	□ più di due volte
Volontariato	□ mai	□ uno o due volte	□ più di due volte
Leggere (Libri)	□ mai	□ uno o due volte	□ più di due volte
Guardare la TV	□ mai	□ uno o due volte	□ più di due volte
Navigare in internet	□ mai	□ uno o due volte	□ più di due volte
Attività manuali per il tempo libero	□ mai	□ uno o due volte	□ più di due volte
(suonare strumenti musicali, dipingere)			
